# Altered Gray Matter Volume, Functional Connectivity, and Degree Centrality in Early-Onset Type 2 Diabetes Mellitus

**DOI:** 10.3389/fneur.2021.697349

**Published:** 2021-09-09

**Authors:** Yue Feng, Yifan Li, Xin Tan, Yi Liang, Xiaomeng Ma, Yuna Chen, Wenjiao Lv, Jinjian Wu, Shangyu Kang, Mingrui Li, Shijun Qiu

**Affiliations:** ^1^First Clinical Medical College, Guangzhou University of Chinese Medicine, Guangzhou, China; ^2^Department of Radiology, The First Affiliated Hospital of Guangzhou University of Chinese Medicine, Guangzhou, China

**Keywords:** early-onset type 2 diabetes mellitus, VBM, resting-state fMRI, degree centrality, cognitive performance

## Abstract

**Background:** Structural and functional brain alterations that underlie cognitive decline have been observed in elderly adults with type 2 diabetes mellitus (T2DM); however, whether these alterations can be observed in patients with early-onset T2DM remains unclear. Therefore, we aimed to describe the abnormalities in brain volume and functional patterns in patients with early-onset T2DM in the present study.

**Methods:** We enrolled 20 patients with early-onset T2DM and 20 healthy controls (HCs). Changes in brain volume were assessed using voxel-based morphology (VBM), while changes in brain function were assessed using degree centrality (DC) and functional connectivity (FC).

**Results:** Compared to HCs, patients with early-onset T2DM exhibited gray matter reductions in the left orbital superior, middle, and inferior frontal gyri as well as the right superior frontal gyrus. The gray matter reductions in the right superior frontal gyrus were negatively associated with the urine albumin to creatinine ratio. Furthermore, increased DC values were observed in the left superior temporal gyrus, left Heschl gyrus, and left hippocampus in patients with early-onset T2DM. An FC analysis of these regions revealed elevated connectivity in the right precuneus, left inferior parietal gyrus, left Heschl gyrus, bilateral post-central gyrus, bilateral insula, bilateral superior temporal gyrus, and bilateral medial and paracingulate gyrus. Furthermore, the FC of the hubs to the superior temporal gyrus, insula, and Heschl gyrus was increased and positively correlated with trail making test-B.

**Conclusion:** Decreased local gray matter volume and increased DC and FC may represent the neurobiological mechanism underlying cognitive dysfunction in patients with early-onset T2DM.

## Introduction

Early-onset type 2 diabetes mellitus (T2DM) is diagnosed in those under 40 years of age (1). In adults, an increase in the morbidity of early-onset T2DM has recently been observed worldwide, with an increased prevalence in Asian countries compared to Western countries (1). Therefore, early-onset T2DM can be considered a global epidemic. In 2013, a previous study on the Chinese population revealed that 5.9% of adults aged 18–40 years were diagnosed with diabetes (2). T2DM increasingly affects more individuals of working age leading to the premature development of complications (3, 4). Longitudinal studies (1, 5) have found that patients with early-onset T2DM exhibit poorer diabetic control, an increased risk of retinopathy and microvascular complications, and an increased incidence of microalbuminuria compared to patients with late-onset T2DM. Therefore, despite their younger age and shorter disease course, patients with early-onset T2DM may experience neurological complications such as cognitive dysfunction.

Previous studies have demonstrated brain abnormalities and poor cognitive performance in middle-aged to elderly patients with T2DM (6–9). However, studies regarding brain alterations in patients with early-onset T2DM, particularly in brain hubs, have been lacking. Therefore, we hypothesized that cognitive impairments as well as structural and functional brain abnormalities could be observed in patients with early-onset T2DM. One previous study (10) using voxel-based morphometry (VBM) reported reductions in global gray matter volume (GMV) and regional area in the temporal and occipital lobes in adolescents with T2DM, while another study (11) reported inconsistent results in the left caudate, thalamus, and bilateral amygdala. Moreover, a recent study involving patients under 40 years old found no significant difference in global or voxel-based brain volume (12). Therefore, we aimed to measure the brain volume via VBM in patients with early-onset T2DM.

Degree centrality (DC) is a method of measuring whole-brain connectivity based on the level of voxels, which effectively identifies the brain center. This method has been used in research related to diabetes (13), bipolar disorder (14), and Parkinson's disease (15). DC measures the connectivity of the whole brain to individual regions; this underlies the importance of the local region, termed the “brain hub” (16). Functional connectivity (FC), one of the most common methods in resting-state functional magnetic resonance imaging (fMRI), is defined as functional synchronization between different brain regions, underlying the mechanism of functional integration of distinct brain regions (17). Previous studies (15, 18) have combined the DC and FC to explore the alteration of functional patterns in patients with neurological diseases. A recent study (13) reported increased DC of the right cerebellum and right fusiform gyrus among middle-aged patients with T2DM. Another study (18) with a similar population reported increased DC and FC value of the right anterior insula and dorsal anterior cingulate cortex. However, the changes in the functional patterns of the brain in patients with early-onset T2DM remain unclear. In this study, we aimed to identify the latent neurobiological mechanism of brain alteration for patients with early-onset T2DM by examining alterations in functional patterns, particularly in brain hubs. Furthermore, we analyzed the relationships among altered brain volume, cognitive tests, and T2DM-related biochemical indexes.

## Materials and Methods

### Participants

We recruited patients with early-onset T2DM who consulted at the Department of Endocrinology of the First Affiliated Hospital of Guangzhou University of Chinese Medicine between December 6, 2018, and November 3, 2020 (Table 1). Additionally, a healthy control (HC) group was recruited through advertisements in the local community.

**Table 1:** Demographic and clinical data of all participants.

	**T2DM (** ***n*** **= 20)**	**HC (** ***n*** **= 20)**	**Statistics (t/Z/Fisher)**	***p***
**General and clinical data**				
Age	36.45 ± 3.72	34.05 ± 4.78	1.771	0.085
Gender	14/6	7/13		0.056
Education	12 (9, 14.75)	15.5 (12, 16)	−1.811	0.070
BMI	25.41 ± 3.20	21.91 ± 2.32	3.959	0.000*
Systolic pressure (mmHg)	120.1 ± 16.46	115.6 ± 13.34	0.950	0.348
Diastolic pressure (mmHg)	84.2 ± 8.23	79.5 ± 6.87	2.148	0.038*
Diabetes duration	3.79 ± 0.84	N/A	N.A	N/A
HbA1c (%)	9.80 ± 2.09	N/A	N/A	N/A
FBG (mmol/L)	9.67 ± 3.62	N/A	N/A	N/A
FINS (μIU/ml)	16.25 ± 16.93	N/A	N/A	N/A
TG (mmol/L)	3.21 ± 2.98	N/A	N/A	N/A
TC (mmol/L)	5.11 ± 1.13	N/A	N/A	N/A
LDL (mmol/L)	3.19 ± 1.23	N/A	N/A	N/A
UACR (mg/g)	5.77 (3.55, 8.43)	N/A	N/A	N/A
**Cognitive tests**				
MoCA-B	27 (25.25, 28.75)	28 (26.25, 29.75)	−1.546	0.122
AVLT immediate	24.7 ± 4.14	26.9 ± 4.78	−1.556	0.128
AVLT (5 min)	10.5 (7.25, 11)	11 (9, 11.75)	−1.096	0.273
AVLT (20 min)	10 (7.25, 11)	11 (9, 11.75)	−1.103	0.270
AVLT recognition	12 (9.75, 12)	12 (12, 12)	−1.389	0.165
TMT_A	38 (30, 47.5)	41 (32, 50.5)	−0.542	0.588
TMT_B	29 (26, 48.5)	33 (26.25, 42.75)	−0.014	0.989
GPT (R)	67.5 (60.25, 71)	60 (56, 65.75)	−2.006	0.045*
GPT (L)	72.4 ± 15.15	72.2 ± 14.93	0.042	0.967
SDT	52.8 ± 10.15	61.9 ± 10.29	−2.816	0.008*
DST forward	8 (7.25, 9)	8 (8, 9)	−0.071	0.944
DST backward	5 (5, 7)	5.5 (4, 7.75)	−0.110	0.912
**Brain volume**				
Gray matter volume	656.85 ± 41.77	669.75 ± 66.60	−0.733	0.468
White matter volume	543.71 ± 48.01	550.34 ± 64.41	−0.369	0.714
Cerebrospinal fluid volume	242.01 ± 31.32	213.20 ± 30.59	2.942	0.006*
Total intracranial volume	1,442.57 ± 104.04	1,433.29 ± 146.63	0.231	0.819

*Two-sample t-tests for normalized data, Mann–Whitney tests for non-normalized data, and Fisher's exact tests for sex*.

* *p < 0.05. N/A, not applicable*.

*T2DM, type 2 diabetes mellitus; HC, healthy control; BMI, body mass index; HbA1c, glycosylated hemoglobin; FBG, fasting blood glucose; FINS, fasting insulin; TG, triglyceride; TC, total cholesterol; LDL, low-density lipoprotein; UACR, urinary microalbumin/creatinine ratio; MoCA, Montreal Cognitive Assessment; AVLT, auditory verbal learning test; TMT, trail making test; GPT, grooved pegboard test; SDT, symbol digit test; DST, the digital span test*.

The criteria formulated by the American Diabetes Association (ADA) was used to diagnose T2DM, which is consistent with our previous study (19). The age of diagnosis in the current study is between 18 and 40 years in cases of early-onset T2DM (1, 12). Participants with the following characteristics were excluded: other types of diabetes, organic brain lesions (such as brain trauma, vascular lesions, or tumors), psychiatric disorders, systemic disease(s), and substance abuse. A total of 40 participants were included, of which 20 were included in the HC group and 20 in the early-onset T2DM group. Age, education, and sex were matched between the two groups. Informed consent was obtained from all participants. This research was approved by the Medical Research Ethics Committee of Guangzhou University of Chinese Medicine.

### Clinical Data and Cognitive Tests

We collected data regarding sex, age, educational years, blood pressure (systolic and diastolic blood pressure), and body mass index (BMI). The biochemical tests we performed included triglyceride, total cholesterol, low-density lipoprotein, fasting insulin, fasting blood glucose (FBG), glycosylated hemoglobin (HbA1c), and urine albumin creatinine ratio (UACR) analysis. UACR is an early indicator of microvascular lesions in the kidneys.

All participants participated in a series of cognitive assessments, composed of the Montreal Cognitive Assessment (MoCA, Beijing edition) (20), trail making test (TMT) (21), grooved pegboard test (GPT; involving both right- and left-handed) (22), symbol digit test (SDT) (23), the digit span test (DST, involving forward and backward) (24), and the auditory verbal learning test (AVLT) (25). The MoCA is used to identify the subjects with cognitive impairment, while the TMT is used to measure visuospatial function and executive function by comparing the time it takes to accomplish the tests, including parts A and B; the GPT reflects motor function and executive function, while the SDT is used to measure the working memory and attentive function. The results of DST can reflect the working memory of the participants. The AVLT is used to measure the participants' memory function by calculating the number of words remembered over a specified time and usually involves four parts: immediate memory tests, 5-min delayed tests, 20-min delayed tests, and recognition. Participants spent at least 30 min on these evaluation procedures.

### Image Acquisition

Magnetic resonance imaging (MRI) data were obtained using a 3.0T SIEMENS MAGNETOM Prisma MRI scanner with a 64-channel phased-array head coil. We obtained conventional sequences for screening from T1-weighted and fluid-attenuated inversion recovery (FLAIR) images (26). These images were evaluated by two experienced radiologists. Experimental sequences also covered structural and functional sequences. Structural images were obtained using magnetization-prepared rapid gradient echo (MPRAGE) sequences with the parameters calibrated as follows: Repetition time (TR) = 2,530 ms; echo time (TE) = 2.98 ms; matrix acquisition = 256/256; slice thickness = 1 mm; number of slices = 192; flip angle = 7°; field of view (FOV) = 256 mm; voxel size = 1 × 1 × 1 mm. On the other hand, functional images were obtained using a simultaneous multi-slice technique. The scanning parameters were calibrated as follows: TR = 500 ms; TE = 30 ms; flip angle = 60°; number of slices = 35; FOV = 224 mm; slice thickness = 3.5 mm; accelerating factor = 1; voxel size = 3.5 × 3.5 × 3.5 mm.

### Voxel-Based Morphometry Analysis

The VBM toolbox (version 445, http://dbm.neuro.uni-jena.de/vbm/) was used with the default parameters running statistic parametric mapping (SPM8) software (http://www.fil.ion.ucl.ac.uk/spm/). The 3D-T1 images of each participant were segmented and then spatially registered using the DARTEL method. The processed images were further smoothed using an 8-mm full width at half-maximum (FWHM). Finally, the brain volumes of the gray matter (GM), white matter (WM), and cerebrospinal fluid were obtained, while we calculated the total intracranial volume simultaneously.

### Preprocessing of Resting-State fMRI Data

The original data were preprocessed on the MATLAB R2013a platform using the Data Processing Assistant for Resting-state fMRI (version 4.1, http://www.restfmri.net/forum/DPARSF) (27). To reduce the influence of unstable factors, we deleted the first 10 images, while the remaining 950 images were processed. Additionally, conducting slice timing during preprocessing was not necessary for images with lower-echo time (28). Participants with head motion exceeding 2 mm in translation or 2° in rotation were excluded; however, none were excluded in this step. The images were then spatially registered to the standard Montreal Neurological Institute (MNI) space, followed by detrending, regressing, and bandpass filtering (0.01–0.08 Hz).

### DC Analyses and Seed-Based FC Analyses

DC values were calculated using the DPARSF toolbox with a threshold of *r* > 0.25 (18). We calculated the weighted DC values as the number of connected edges in the current study (29). The z transformation was displayed to improve normality. The DC values of each participant were further smoothed with a 6-mm FWHM.

The peak MNI coordinates of brain regions with altered DC values were considered as the center of the spherical seed within a 6-mm radius. The spherical region of interest was selected as the seed to calculate FC values. The average time course of each area was calculated. Finally, correlation coefficients were converted to z scores using Fisher r-to-z transformation to improve normality.

### Statistical Analysis

Statistical analyses of the general data were performed using the SPSS software (Version 26.0; IBM, Armonk, New York). Firstly, the Shapiro–Wilk test was applied to test the normality of the general data. The two-sample *t*-test was then used to analyze the normalized data; on the other hand, we applied the Mann–Whitney non-parametric tests for non-normalized data. The Fisher's exact test was performed to identify differences based on sex. Furthermore, the two-sample *t*-test was conducted using the SPM 12 software (http://www.fil.ion.ucl.ac.uk/spm/) to compare brain volume, DC, and FC maps between the two groups. Age, sex, education, and diastolic blood pressure were set as covariates when we compared brain volume, DC, and FC maps. The AlphaSim correction was displayed at a *p* < 0.05 (5,000 simulations, voxel *p* = 0.001) for VBM analysis, while cluster-level familywise error (FWE) correction (*p* < 0.05) was executed for DC and FC analysis. In addition, the partial correlation method was performed to analyze the association among imaging indexes (results of DC and FC analysis), biochemical indexes, and cognitive data of the patients with age, sex, and education as covariates. The statistical significance was set at *p* < 0.05.

## Results

### Clinical Data and Neuropsychological Tests

No organic lesions or white matter hyperintensities (WMHs) were observed in the conventional images of the participants; therefore, none were excluded based on this criterion. The general, clinical, and cognitive information of the two groups is shown in Table 1. Patients with early-onset T2DM had higher BMI (*p* = 0.001), and diastolic pressure (*p* = 0.038) than HCs. GPT-R (*p* = 0.045) and SDT (*p* = 0.008) scores were significantly lower in the early-onset T2DM group than in the HCs. Other measured parameters showed no significant difference between the two groups.

### Brain Volume

**Figure 1:**
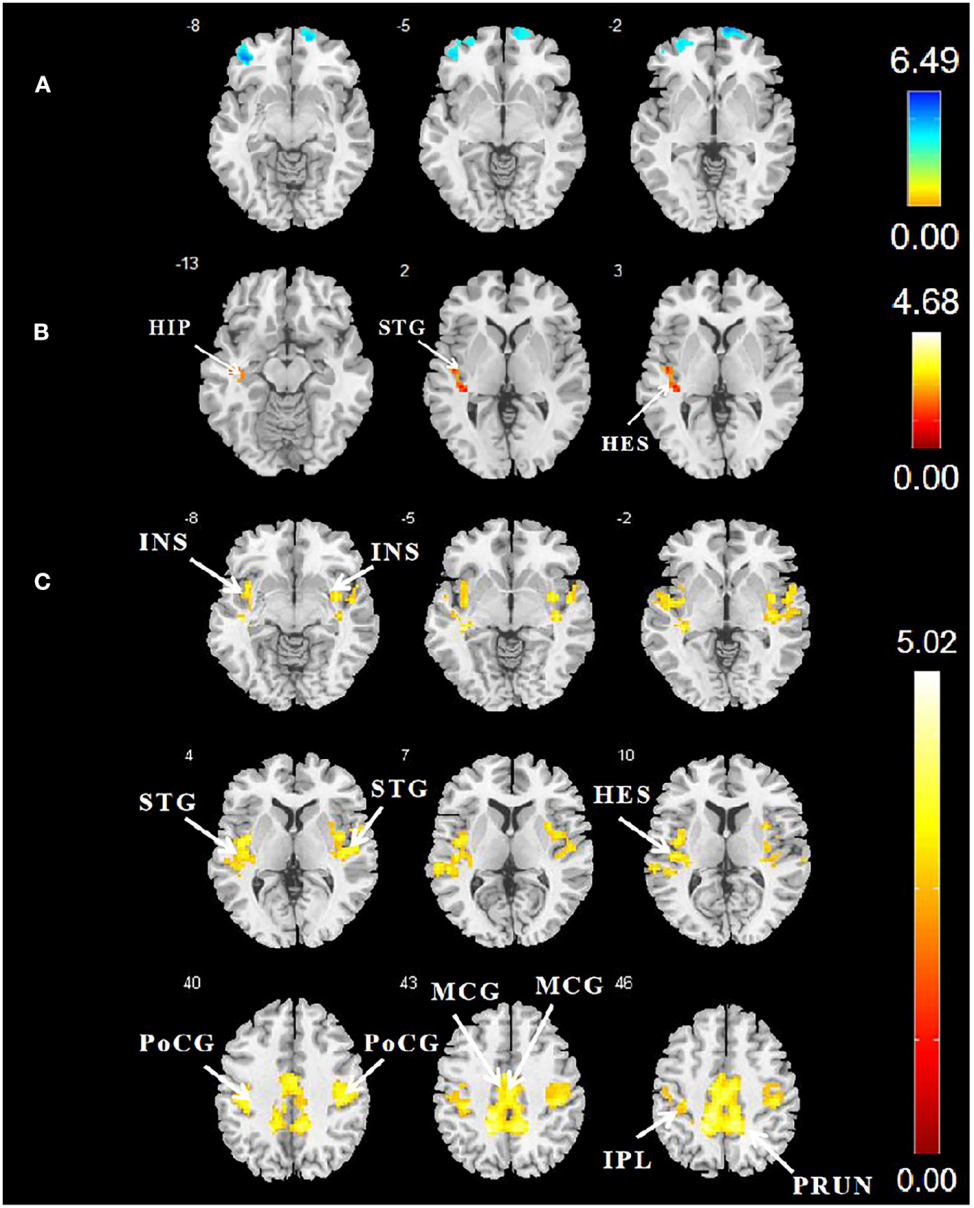
**(A)** Differences in GMV between patients with early-onset T2DM and HCs. AlphaSim correction (5,000 simulations, voxel *p* = 0.001, and cluster size ≥ 586 voxels). **(B)** Comparison of DC values between the two groups. Cluster-level FWE correction (*p* < 0.05, cluster size ≥ 80 voxels). **(C)** Comparison of FC values between the two groups. Cluster-level FWE correction (*p* < 0.05, cluster size ≥ 90 voxels). The color bar denotes the *t*-value. GMV, gray matter volume; T2DM, type 2 diabetes mellitus; HCs, healthy controls; DC, degree centrality; FWE, familywise error; FC, functional connectivity.

**Figure 2:**
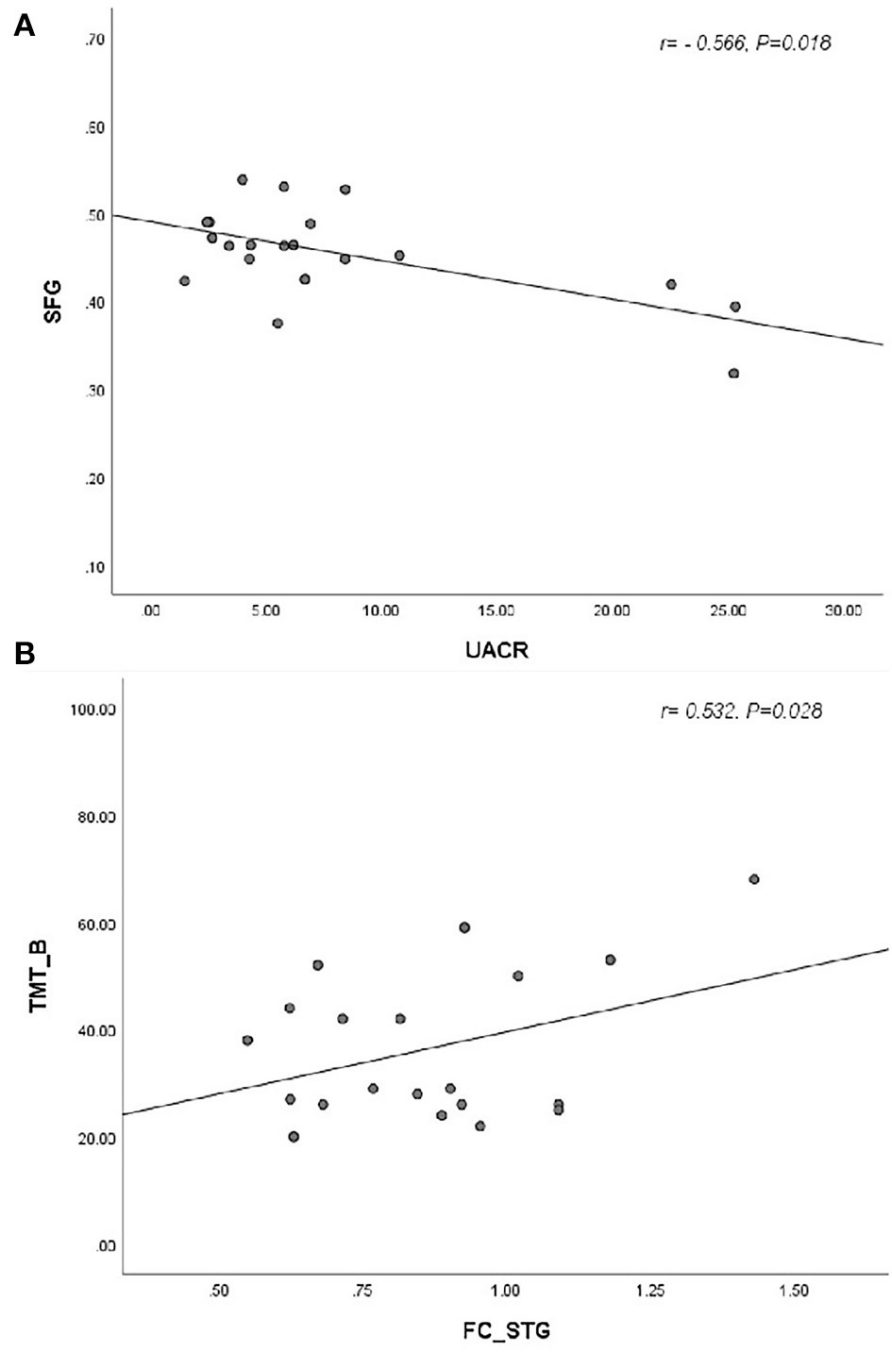
**(A)** The reduced gray matter volume in SFG was negatively associated with the UACR (*r* = −0.566, *p* = 0.018). **(B)** The functional connectivity in STG, insula, and postcentral gyrus was positively correlated with TMT-B (*r* = 0.532, *p* = 0.028). SFG, superior frontal gyrus; UACR, urine albumin creatinine ratio; STG, superior temporal gyrus; TMT-B, trail-making test-B].

According to the two-sample *t*-test in SPSS, the early-onset T2DM group had an elevated mean cerebrospinal fluid volume. There were no significant differences between two groups in terms of total GMV and WM volume and total intracranial volume (Table 1). After adjusting for age, sex, education, and diastolic blood pressure, two clusters with decreased GMV survived in the early-onset T2DM group when compared to the HC group, including the left orbital superior, middle, and inferior frontal gyri, as well as right orbital, medial, and intraorbital superior frontal gyri (SFG). No brain region demonstrated an increase in GMV (Table 2 and Figure 1A). Additionally, decreased volume of the SFG was negatively correlated with UACR, after controlling for age, sex, and education (Figure 2A).

**Table 2:** Comparison of GMV, DC, and FC between early-T2DM and HC groups.

**Cluster**	**Regions**	**Peak MNI**			**Number of voxels**	**t-value**
		**X**	**Y**	**Z**		
**VBM**						
Cluster 1	Frontal_Sup_R	12	70.5	3	172	−5.1108
	Frontal_Sup_Orb_R				169	
	Frontal_Mid_Orb_R				128	
Cluster 2	Frontal_Mid_Orb_L	−39	42	−9	241	5.0316
	Frontal_Inf_Orb_L				109	
	Frontal_Sup_Orb_L				102	
**Degree centrality**						
Cluster 1	L_STG	−42	−21	−12	22	5.6034
	L_HES				11	
	L_HIP				3	
**Functional connectivity**						
Cluster 1	R_PoCG	39	−15	0	122	5.1308
	R_STG				111	
	R_INS				79	
Cluster 2	L_STG	−39	−30	−6	117	5.5411
	L_INS				90	
	L_HES				39	
Cluster 3	L_MCG	−15	−48	42	239	6.1663
	R_MCG				200	
	R_PRUN				168	
Cluster 4	L_PoCG	−36	−27	39	65	4.8955
	L_IPL				18	

*GMV, gray matter volume; DC, degree centrality; FC, functional connectivity; T2DM, type 2 diabetes mellitus; HC, healthy control; MNI, Montreal Neurological Institute; VBM, voxel-based morphology; Frontal_Sup_Orb_R, right orbital superior frontal gyrus; Frontal_Sup_R, right superior frontal gyrus; Frontal_Mid_Orb_R, right medial orbital superior frontal gyus; Frontal_Mid_Orb_L, left orbital middle frontal gyrus; Frontal_Inf_Orb_L, left orbital inferior frontal gyrus; Frontal_Sup_Orb_L, left orbital superior frontal gyrus; STG, superior temporal gyus; PoCG, postcentral gyrus; INS, insular; HES, Heschl; MCG, midcingulate gyrus; IPL, inferior parietal lobule; PCUN, precuneus; HIP, hippocampus*.

### Degree Centrality

Since weighted and binarized results were similar, weighted maps were finally used to calculate the DC values. Compared with the HC group, patients with early-onset T2DM had one cluster survived with increased DC in the left superior temporal gyrus (STG), left Heschl gyrus, and left hippocampus (Table 2 and Figure 1B) after controlling for age, sex, education, and diastolic blood pressure. The thresholds of 0.2 and 0.4 were used to verify the reliability of the results (Supplementary Tables 1 and 2).

### Functional Connectivity

After controlling for age, sex, education level, and diastolic blood pressure, patients with early-onset T2DM exhibited increased FC values primarily in the right precuneus, left inferior parietal gyrus, left Heschl gyrus, bilateral postcentral gyri (PoCG), bilateral STG, bilateral insula, and bilateral midcingulate gyri (MCG) (Table 2 and Figure 1C). Furthermore, increased FC of the hubs to the STG, insula, and PoCG were positively correlated with TMT-B after adjusting for age, education, and sex (Figure 2B).

## Discussion

In our study, we used VBM and rs-fMRI to elucidate the mechanisms underlying cognitive disorders in patients with early-onset T2DM. Firstly, the VBM analysis revealed lower GMV primarily in the frontal gyrus, and lower GMV in the SFG was associated with UACR, which underlies cognitive dysfunction. Additionally, increased DC values were observed in the STG, Heschl gyrus, and hippocampus. Finally, we observed extensive changes in hub-based FC values in the precuneus, Heschl gyrus, inferior parietal gyrus, STG, MCG, and PoCG, and FC from hubs to STG, insula, and PoCG may be associated with impaired working memory.

### GMV Change

We found lower GMV in patients with early-onset T2DM when compared to HCs primarily in the left orbital superior, middle, and inferior frontal gyri, as well as the right orbital, medial, and intraorbital SFG.

Our findings of superior/middle/inferior frontal gyri atrophy in early-onset T2DM were consistent with those of previous studies (6, 30). Cognitive decline in patients with diabetes may be caused by hyperglycemia and insulin resistance, as these are known to induce neuroinflammation through the oxidative stress pathways (31, 32). Chronic hyperglycemia and oxidative stress may result in the dysregulation of the internal environment of the cerebral vasculature, resulting in structural changes of the affected areas. The findings of previous studies have demonstrated that patients with early-onset T2DM have lower GMV (33, 34), GM density (9, 32), and cerebral glucose metabolism (9). A previous study (32) on middle-aged to elderly adults also reported the association of reduced prefrontal cortex density with cognitive dysfunction and poor glycemic control, which is consistent with our findings. Additionally, the SFG is a region located in the prefrontal cortex that connects other brain regions (35). Both the impairments in frontal fiber tracts and activation of the SFG were correlated with poor working memory (8, 36, 37). However, we observed no association between lower GMV in the frontal gyrus and cognitive performance of the participants in our study. The young adults we recruited had overall greater educational attainment than the elderly patients, who might have relatively high cognitive reserve which could compensate for the diminished cognitive function. One previous study (38) demonstrated lower brain volumes and higher brain function in patients with mild cognitive impairment or Alzheimer's disease who have higher cognitive reserve. Thus, reduced GMV in patients with early-onset T2DM may underlie more advanced neuropathology; however, the mechanism remains unclear. In contrast to our study on patients with relatively long course of T2DM, one previous study (12) on similar T2DM group without diabetic complications did not demonstrate a significant difference in global and regional brain volume between groups of T2DM and HCs, which may demonstrate different brain structural changes at different stages of T2DM progression.

Furthermore, we observed that UACR was negatively associated with the GMV in the right SFG, which is consistent with some previous studies. Clinically, higher prevalence rates of retinopathy and albuminuria are observed in patients with early-onset T2DM (39). Longitudinal studies have demonstrated the correlation of UACR with severe diabetes-related consequences and mortality (40, 41). Additionally, it was reported that the UACR was associated with executive function and brain structural alterations in elderly adults with T2DM (41). Furthermore, a Maastricht study (42) indicated that albuminuria was associated with cognitive dysfunction and reduced information processing speed among the elderly adults. Therefore, the results of this study indicate that UACR may be an early index for the evaluation of changes in brain volume in patients with early-onset T2DM.

### DC Alteration

DC can be used to measure the significance of the neural activity of one region in the brain in relation to the whole brain. In the present study, brain areas with high DC values were defined as brain hubs. We observed increased DC values in the left STG, left Heschl gyrus, and left hippocampus in our study (16). Previous studies have reported the critical role of the temporal lobe and hippocampus in diabetic-related changes (43, 44). Additionally, the STG is related to auditory function and short-term memory (45). The results of a previous study (44) suggested potential compensatory mechanisms of the temporal gyrus by demonstrating changes in the amplitude of low frequency fluctuation in the temporal gyrus of patients with T2DM and mild cognitive impairment compared to those with only mild cognitive impairment. Accumulated evidence suggests the hippocampus plays a key role in declarative or memory function (46). Previous literature (47) demonstrated an abnormal activation in the hippocampus of patients with T2DM which was associated with memory function. Furthermore, a recent study demonstrated that a correlation between the hippocampus and olfactory memory may exist (48). Therefore, increased DC values in the temporal gyrus and hippocampus might be correlated with impairment of memory function. However, we did not observe significant differences between increased DC values and cognitive tests, which could be attributed to the compensation mechanism.

### Hub-Based Functional Connectivity Alteration

Analysis of resting-state FC is essential for better understanding of the brain functional patterns in the brain. Through FC calculation based on DC analysis, we found increased FC of hubs to the right precuneus, left inferior parietal gyrus, left Heschl, bilateral PoCG, bilateral STG, bilateral insula, and bilateral MCG in patients with early-onset T2DM. In addition, the increased FC of the hubs to the STG, insula, and Heschl gyrus was negatively related to memory function. A previous study has stated that increased FC underlies a compensatory mechanism to offset neural deterioration and cognitive decline (49). Another explanation suggests that increased FC is a pathological state that results in brain impairment (50). In the present study, we observed a positive correlation between increased FC and TMT-B; therefore, we speculate that increased FC underlies pathological changes but that compensatory mechanisms are still at work.

Firstly, the STG and Heschl gyrus are part of the auditory cortex and associated with auditory function. Similar regions were reported in DC and FC in the STG and Heschl gyrus region, indicating a connection between these regions. One previous study found biochemical changes of the auditory cortex in women with T2DM, suggesting a possible complication related to mental disorders in patients with T2DM. In addition, increased FC of hubs to the STG, insula, and Heschl gyrus were found to be positively correlated with TMT-B which is mainly associated with working memory and inhibition control. The longer time it takes for patients to accomplish the test, the worse memory function participants may have. One previous report (51) identified a fiber tract between the insula and STG. Our results indicate that there may be an association between this connection and memory function. Additionally, it is demonstrated that an association exists between STG and auditory short-term memory. The insula is related to advanced cognitive processes. Both STG and insula may be involved in memory formation. The increased FC may underlie potential compensation for slight cognitive disorders by the recruitment of other brain regions. Furthermore, one previous study (52) found that patients with T2DM had reduced GMV in the STG and insula. Another previous study (53) observed reduced FC values between the insula and temporal regions associated with hypoglycemia in patients with type 1 diabetes mellitus (T1DM). The mechanisms of cognitive dysfunction of T1DM and T2DM are similar despite differences in pathophysiological mechanisms. However, we found that no specific brain region was correlated with HbA1c or FBG. We may have insufficient evidence to observe a relationship due to the small samples of MRI and biochemical tests.

Secondly, the precuneus and the inferior parietal gyrus are crucial nodes in the default mode network. The precuneus is located in the posterior medial part of the parietal lobe and plays a vital role in the integration of higher-order cognitive function, including visuo-spatial imagery and episodic memory. As a key node in default mode network, the inferior parietal gyrus also plays a crucial part in memory and executive function. Previous studies have found various functional changes in this area in the group with T2DM. Huang et al. (8) reported the activation of the bilateral precuneus and parietal area in middle-age patients with T2DM during 2-back task. Duan et al. (54) observed decreased cerebral blood flow in precuneus area. Xiong et al. (55) demonstrated elevated nodal characteristics in the parietal region. In addition, there are fiber tracks between STG and the precuneus/inferior parietal gyrus area (51). Our findings of increased FC between hubs and the precuneus/inferior parietal gyrus may provide new insights for interpretation of the mechanism of early-onset T2DM. Furthermore, Fang et al. (12) reported increased FC between hippocampus and inferior parietal lobule in a similar group of patients with T2DM, which is similar to our results. However, we observed a larger range of brain regions with increased FC. This might be due to two reasons. Firstly, our results might be more accurate because we evaluated DC to obtain the brain regions (as a seed in FC analysis) with relatively high DC values. Moreover, patients with early-onset T2DM in this study had relatively long disease course and may have had worse cognitive disorders.

Thirdly, the MCG is involved in monitoring conflicts and decision-making (56). A previous study found structural or functional abnormalities in the medial and paracingulate gyrus in patients with T2DM (57). However, the mechanisms underlying these abnormalities remain unclear. One study (58) found that the FC of the ventral striatum to the insula and midcingulate cortex was associated with diabetes and depression. Additionally, the anterior midcingulate cortex is also often described in the literature as the dorsal anterior cingulate cortex (59). In a task-state MRI study (60), both decreased and increased activation of the anterior cingulate cortex were reported in the T2DM group (7, 60). One previous study (18) reported increased FC and DC values of the right insula and dorsal anterior cingulate cortex which constitute crucial parts of the salience network related to higher-order cognitive processes, similar to some of our results. However, no association was found between the MCG and cognitive tests. A small sample size may limit the effect, and more subjects are required for further studies. The PoCG comprises the primary somatosensory cortex which may help integrate sensorimotor function. Previous studies have shown abnormal activation and reduced cortical thickness of the primary somatosensory cortex in patients with T2DM (61, 62). The mutual effect of motor and cognitive function has been established. There is evidence that cortical changes in the motor area affects the hand dexterity of people with diabetes (63). Additionally, slow gait speed was reported to be an early index of cognitive dysfunction in the elderly adults (64). Therefore, increased FC in the PoCG might indicate the potential impairment of sensorimotor and cognitive function in patients with early-onset T2DM (62).

Our study had several limitations. First, we recruited a relatively small sample size which may have affected the results and generalizability of the study. Second, the partial correlation analysis did not allow for multiple corrections, which weakened the basis of our inference. Third, the cross-sectional design was unable to reveal causal relationships or the progression of structural and functional changes. Therefore, future studies should include larger samples and employ longitudinal designs.

## Conclusion

Our preliminary study on patients with early-onset T2DM demonstrated GMV loss and increased DC and FC values in these patients. We observed reduced GMV in the bilateral frontal gyri, primarily in the right SFG, which may be related to changes in cognitive function in patients with T2DM. In addition, the increases in the FC of hubs to the STG, Heschl gyrus, and insula might be negatively associated with memory function. In summary, our results indicate that structural and functional brain changes may indicate potential neurological mechanisms for cognitive disorders in patients with early-onset T2DM. Further investigation is necessary to verify the findings of our study.

## Data Availability Statement

The raw data supporting the conclusions of this article will be made available by the authors, without undue reservation.

## Ethics Statement

The studies involving human participants were reviewed and approved by Medical Research Ethics Committee of Guangzhou University of Chinese Medicine. The patients/participants provided their written informed consent to participate in this study.

## Author Contributions

YF, XM, JW, WL, SK, and ML participated in the process of recruitment, MRI scanning, and cognitive assessments. XT and YLia were responsible for the inspection of the images and diagnosis. YF finished the data processing and the manuscript with the assistance of WL, YC, and XM. YLi and XT guided the revision of the manuscript. SQ contributed to the coordination and cooperation between the clinical department and imaging department, and was in charge of the inspection of the whole study. All authors contributed to the article and approved the submitted version.

## Funding

This work was supported by the grants from the Key International Cooperation Project of National Natural Science Foundation of China (81920108019) and the Medical Scientific Research Foundation of Guangdong Province (A2021182).

## Conflict of Interest

The authors declare that the research was conducted in the absence of any commercial or financial relationships that could be construed as a potential conflict of interest.

## Publisher's Note

All claims expressed in this article are solely those of the authors and do not necessarily represent those of their affiliated organizations, or those of the publisher, the editors and the reviewers. Any product that may be evaluated in this article, or claim that may be made by its manufacturer, is not guaranteed or endorsed by the publisher.
