# Supplementary material for: Altered Gray Matter Volume, Functional Connectivity, and Degree Centrality in Early-Onset Type 2 Diabetes Mellitus
Source: Front Neurol. 2021 Sep 9;12:697349. doi: 10.3389/fneur.2021.697349 (PMC8459017; doi:10.3389/fneur.2021.697349)
Supplement: Supplementary file 1 [file Data_Sheet_1.docx]

Supplementary Material

# Supplementary Figures and Tables

## Supplementary Tables

**Supplementary Table 1.** Degree centrality with threshold at 0.4

| Cluster | regions | Peak MNI | | | number of voexls | t value |
| --- | --- | --- | --- | --- | --- | --- |
|  |  | X | Y | Z |  |  |
| Cluster1 | L_STG | -42 | -21 | -12 | 33 | 5.9988 |
|  | L_HES |  |  |  | 18 |  |
|  | L_HIP |  |  |  | 8 |  |

*STG, superior temporal gyrus; HIP, Hippocampus; HES, Heschl gyrus*

**Supplementary Table 2.** Degree centrality with threshold at 0.2

| Cluster | regions | Peak MNI | | | number of voexls | t value |
| --- | --- | --- | --- | --- | --- | --- |
|  |  | X | Y | Z |  |  |
| Cluster1 | L_STG | -42 | -21 | -12 | 19 | 6.0534 |
|  | L_HES |  |  |  | 8 |  |
|  | L_MTG |  |  |  | 2 |  |

*STG, superior temporal gyrus; HES, Heschl gyrus; MTG, middle temporal gyurs*
